# MicroRNAs Deregulated in Intraductal Papillary Mucinous Neoplasm Converge on Actin Cytoskeleton-Related Pathways That Are Maintained in Pancreatic Ductal Adenocarcinoma

**DOI:** 10.3390/cancers13102369

**Published:** 2021-05-14

**Authors:** Elena Fernandez-Castañer, Maria Vila-Casadesus, Elena Vila-Navarro, Carolina Parra, Juan Jose Lozano, Antoni Castells, Meritxell Gironella

**Affiliations:** 1 Gastrointestinal & Pancreatic Oncology Group, Centro de Investigación Biomédica en Red de Enfermedades Hepáticas y Digestivas (CIBERehd)/Hospital Clínic of Barcelona/Institut d’Investigacions Biomèdiques August Pi i Sunyer (IDIBAPS), University of Barcelona, 08036 Barcelona, Catalonia, Spain; EFERNANDEZC@clinic.cat (E.F.-C.); elena.vila@gruposolti.org (E.V.-N.); carolina.parra@ciberehd.org (C.P.); castells@clinic.cat (A.C.); 2 Bioinformatics Platform, Centro de Investigación Biomédica en Red de Enfermedades Hepáticas y Digestivas (CIBERehd), 08036 Barcelona, Catalonia, Spain; mvila@vhio.net (M.V.-C.); juanjo.lozano@ciberehd.org (J.J.L.)

**Keywords:** pancreatic cancer, pancreatic cyst, premalignant lesion, cancer progression, gene regulation, cell structure, miR-181a, stress fibers, circumferential actomyosin belt

## Abstract

**Simple Summary:**

Understanding early events occurring during Pancreatic Ductal Adenocarcinoma (PDAC) establishment can be of huge relevance for improving its dramatic clinical outcome. Intraductal Papillary Mucinous Neoplasms (IPMN) are pancreatic duct lesions associated with increased risk of developing PDAC. MicroRNAs (miRNAs) are small non-coding RNAs significantly altered in IPMN and PDAC that repress mRNA expression. In this study, we have explored negative correlations between miRNA and mRNA expression data in IPMN that are maintained in PDAC, to purpose potential miRNA-mRNA pairs. Relevant negative correlations have been validated via RT-qPCR in an independent cohort of 40 patients. In-situ hybridization of top-correlated miR-181a and immunohistochemistry of its purposed pairs (EPB41L4B and SEL1L), plus validation in a cellular model of immortalized human pancreatic epithelial ductal cells overexpressing this miRNA has been performed. Overall, these results offer new insights into the implications of miRNA alteration in the IPMN-to-PDAC progression.

**Abstract:**

Intraductal papillary mucinous neoplasms (IPMN) are pancreatic cystic lesions that can develop into pancreatic ductal adenocarcinoma (PDAC). Although there is an increasing incidence of IPMN diagnosis, the mechanisms of formation and progression into invasive cancer remain unclear. MicroRNAs (miRNAs) are small non-coding RNAs, repressors of mRNA translation, and promising diagnostic biomarkers for IPMN and PDAC. Functional information on the role of early-altered miRNAs in this setting would offer novel strategies for tracking the IPMN-to-PDAC progression. In order to detect mRNAs that are likely to be under miRNA regulation in IPMNs, whole transcriptome and miRNome data from normal pancreatic tissue (*n* = 3) and IPMN lesions (*n* = 4) were combined and filtered according to negative correlation and miRNA-target prediction databases by using miRComb R package. Further comparison analysis with PDAC data allowed us to obtain a subset of miRNA-mRNA pairs shared in IPMN and PDAC. Functional enrichment analysis unravelled processes that are mainly related with cell structure, actin cytoskeleton, and metabolism. MiR-181a appeared as a master regulator of these processes. The expression of selected miRNA-mRNA pairs was validated by qRT-PCR in an independent cohort of patients (*n* = 40), and then analysed in different pancreatic cell lines. Finally, we generated a cellular model of HPDE cells stably overexpressing miR-181a, which showed a significant alteration of actin cytoskeleton structures accompanied by a significant downregulation of EPB41L4B and SEL1L expression. In situ hybridization of miR-181a and immunohistochemistry of EPB41L4B and SEL1L in pancreatic tissues (*n* = 4 Healthy; *n* = 3 IPMN; *n* = 4 PDAC) were also carried out. In this study, we offer insights on the potential implication of miRNA alteration in the regulation of structural and metabolic changes that pancreatic cells experience during IPMN establishment and that are maintained in PDAC.

## 1. Introduction

Pancreatic ductal adenocarcinoma (PDAC) is currently the seventh leading cause of cancer-related deaths worldwide and the fourth in Europe and United States, with a five-year survival rate of only 10% [1,2]. Intraductal papillary mucinous neoplasms (IPMN) are cystic neoplasms of the pancreas that can lead to cancer either concomitantly or after resection, and they are considered a risk factor for PDAC development [3]. They harbour genomic, transcriptomic, and epigenomic aberrations that are related with the gain of potentially malignant characteristics. Evidences support that IPMN arise from ductal epithelial cells [4], and can progress to PDAC through a range of dysplastic changes [5,6]. High-throughput sequencing techniques have substantially increased our knowledge on IPMN identity, but the mechanisms underlying its establishment and progression into PDAC are yet unclear.

MicroRNAs (miRNAs) are small non-coding RNAs that negatively regulate gene expression at the post-transcriptional level by specific targeting of mRNAs. MiRNAs can modulate the expression of more than 60% of protein-coding genes, generating complex networks [7]. Specific interactions between miRNAs and mRNAs are endogenously tuning a wide range of biological processes altered during tumour initiation and progression, such as cellular differentiation, metabolism, or epithelial-to-mesenchymal transition, in the context of pancreatic neoplasia [8].

IPMN lesions show specific mRNA and miRNA expression profiles that share some commonalities with PDAC [9,10]. The detection of deregulated miRNAs in IPMN or PDAC has been demonstrated to be a potentially successful strategy for the early detection and prevention of the disease [11]. Nevertheless, insights on the global role of miRNA alterations in this context are still lacking.

Although there have been attempts to undertake functional integrative analyses of miRNA-mRNA interactions in different cancer types [12], the characteristics of miRNA-mRNA networks in precancerous lesions, and their contribution to the tumoral setting, are elusive. Detailed information on miRNA-mRNA interaction networks in IPMN, and its presence or not in PDAC, would highlight miRNA-regulated pathways that might be relevant for understanding tumour establishment and malignisation.

In this study, we integrate miRNome and transcriptome paired expression data from IPMN samples with a previously reported software, called miRComb [13], in order to decipher the miRNA-mRNA network in IPMN and describe the functional programmes in which they are involved. MiRNA-mRNA interaction data from our previous study in PDAC [14] are further used for comparative analysis, allowing us to identify a set of miRNA-regulated programmes in IPMN that are maintained during progression to PDAC. Expression analysis in an independent cohort of patients plus cellular studies have been carried out in order to validate the potential functional correlations between revealed miRNA-mRNA pairs, unravelling specific pathways that are being tuned by miRNAs during IPMN-to-PDAC progression.

## 2. Materials and Methods

### 2.1. Clinical Samples

An initial set of seven surgical pancreatic tissue samples (four IPMN and three Healthy) that were taken from patients of the Hospital Clínic of Barcelona (Barcelona, Spain) was used for both miRNA and mRNA profiling. Pancreatic tissues were kept on dry ice during handling, flash frozen in liquid nitrogen, and stored at −80 °C until RNA isolation. Healthy pancreatic samples correspond to the adjacent non-tumoral part of IPMN lesions or from patients who underwent surgery for other reasons (i.e., ampulloma or neuroendocrine tumours). IPMN samples were taken from benign cysts.

The validation of candidate miRNA-mRNA couples was done by qRT-PCR in an independent set of 40 surgical samples (14 PDAC, nine IPMN, 17 Healthy) from individuals that were prospectively included in Hospital Clínic of Barcelona between 2008 and 2013. Supplementary Table S1 shows clinical information related to these patients.

This study was approved by the Institutional Ethics Committee of Hospital Clínic of Barcelona (March 27, 2008) and written informed consent was obtained from all patients in accordance with the Declaration of Helsinki.

### 2.2. RNA Extraction

The total RNA, including miRNA, was isolated from frozen macro-dissected tissues using the MiRNeasy Mini Kit (Qiagen, Valencia, CA, USA), according to the manufacturer protocol. RNA concentrations and purity were evaluated using NanoDrop 1000 Spectrophotometer (Wilmington, DE, USA), and RNA quality was determined by Bioanalyzer 2100 (Agilent, CA, USA).

### 2.3. MiRNome and Transcriptome Data Obtention

Genome-wide miRNA profiling was done by next generation sequencing (NGS) technology on a Genome Analyzer IIx (Illumina, CA, USA), as described in our previous study [9]. Matched genome-wide mRNA profiling was analysed by microarray technology with Human Genome U219 Gene Expression Arrays (Affymetrix, Santa Clara, CA, USA). MiRNome and transcriptome paired data were processed, as described in our previous study [14] using R CRAN and LIMMA.

### 2.4. Integrative Analysis

MiRNA–mRNA interactions were calculated using miRComb R package [14]. Briefly, Pearson correlation coefficients between the expression of a particular miRNA and all of the expressed mRNAs in the same sample were computed and matched with target prediction information using three databases: targetScan_v7.1_17 (http://targetscan.org, accessed on 7 November 2017), miRSVR_aug10_17 (http://www.microrna.org/microrna/home.do, accessed on 7 November 2017), and miRDB_v5.0_21 (http://mirdb.org/miRDB, accessed on 7th November 2017). The miRNA-mRNA obtained pairs were filtered, selecting those that show significant differential expression (adjusted *p*-value < 0.05), negative correlation, and were predicted in at least one of the used databases.

For the enrichment analysis, KEGG and GO databases were applied with miRComb R package (which implements the hypergeometric test) and enrichment comparisons were done using clusterProfiler software [15]. The networks were constructed and visualized with Cytoscape version 3.7.2 (http://www.cytoscape.org, accessed on 23 June 2020). *P*-values from the Pearson correlation estimates were corrected for multiple testing using the Benjamini–Hochberg method.

### 2.5. Analysis of RNA Expression by qRT-PCR

The validation of mRNA and miRNA expression was assessed by qRT-PCR using TaqMan mRNA and miRNA Assays (Thermo Fisher Scientific Inc., Foster City, CA, USA). Analysis included a two-step protocol involving RT, with a RNA-specific primer in the case of miRNA analysis, followed by a real-time PCR with TaqMan. Briefly, 25 ng or 5 ng of total RNA was used in each RT reaction, for mRNA or miRNA evaluation, respectively, and cDNA was analysed using a Viia7 Real-Time PCR System (Thermo Fisher Scientific Inc.). An additional preamplification step using TaqMan preamp Master Mix Kit (Thermo Fisher Scientific Inc.) was applied for miR-372 analysis due to low basal levels. RNU6B or PPIA were used as endogenous controls for miRNA and mRNA analysis, respectively. The Ct values were calculated using 0.1 thresholds. No template controls showed any amplification.

### 2.6. In Situ Hybridization and Immunohistochemistry

For in situ hybridization analysis of miR-181a-5p expression in pancreatic human samples (*n* = 4 Healthy, *n* = 3 IPMN and *n* = 4 PDAC formalin fixed paraffin embedded pancreatic tissues), a 5′- and 3′-double digoxigenin-labeled miRCURY LNA microRNA Detection Probe (Exiqon, Qiagen, Vedbaek, Denmark) was used following the manufacturer’s protocol. The scrambled probe was included as control. Briefly, the deparaffinized, proteinase K-digested sections were hybridized with 60  nM double-DIG LNA hsa-miR-181a-5p probe overnight at 50  °C. The sections were stringently washed with saline-sodium citrate buffer at 50 °C, blocked, and then incubated for 1h with 1:200 dilution of anti-DIG Fab fragments that were conjugated to alkaline phosphatase (Roche Diagnostics, Indianapolis, IN, USA). The signal was detected by incubation with freshly prepared NBT/BCIP AP substrate (Roche Diagnostics) for 2 h at 30  °C. The slides were counterstained with Nuclear Fast Red (Vector laboratories, Burlingame, CA, USA).

Immunohistochemical staining was also carried out in the above-mentioned pancreatic samples. For EPB41L4B (1/100 dilution; HPA042862, Sigma-Aldrich, St. Louis, MI, USA), antigen retrieval by citrate tampon for 20 min. at room temperature, followed by Bond Polymer Refine Detection, was performed. For SEL1L (1/50 antibody dilution; HPA024267, Sigma-Aldrich), antigen retrieval by citrate tampon for 60 min. at room temperature, followed by Bond Polymer Refine Detection, was performed. The images were taken with an Olympus Bx41 microscope (Olympus America, Tokyo, Japan).

### 2.7. Cell Culture

Immortal human pancreatic duct epithelial HPDE cell line, which was kindly provided by Dr. F.X. Real (CNIO, Madrid, Spain), was cultured and maintained in keratinocyte serum-free (KSF) medium supplemented by epidermal growth factor and bovine pituitary extract. HEK-293T and human pancreatic tumour cell lines PANC-1, BxPC-3, and Capan-2 were obtained from the American Type Culture Collection (ATCC, Manassas, VA, USA). The HEK-293T and PANC-1 cells were maintained in Dulbecco’s Modified Eagle’s Medium (DMEM), BxPC-3 cells were maintained in RPMI 1640 Medium, and Capan-2 cells were maintained in McCoy’s 5A (Modified) Medium, all being supplemented with 10% fetal bovine serum (Gibco, Thermo Fisher Scientific Inc.). The cells were cultured in a humidified atmosphere (5% CO2) at 37 °C and then tested for mycoplasma contamination.

### 2.8. Retrovirus Production and HPDE Infection for Stable Overexpression of miR-181a

Vector miRVec-181a (pMSCV-Blasticidin plasmid) and miRVec-hTR were obtained from the miR-Lib microRNA library (Source Bioscience, Nottingham, UK), and they were transfected into HEK-293TΦ cells with CalPhos mammalian transfection kit (Clontech Laboratories, Takara Bio Company Inc., Mountain View, CA, USA). As a plasmid control, we used miRVec-hTR that contains the sequence for the RNA subunit of the telomerase enzyme, and it is similar in size to the sequence of miRNAs. Virus-containing HEK-293TΦ supernatants were collected at 48h post-transfection, filtered, and used to transduce HPDE cells for constitutive miR-181a overexpression. Twenty-four hours post-infection, HPDE cells were treated with 18 µg/mL blasticidin, for the selection of transduced cells. The expression of miR-181a was assessed via qRT-PCR.

### 2.9. Fluorescence Imaging

In order to detect F-actin, HPDE-hTR-Control and HPDE-miR-181a-5p cells were seeded and attached to glass coverslips. The cells were fixed with 4% paraformaldehyde in PBS for 15 min., permeabilized with 0.1% Triton X-100 for 15  min., and then stained with Rhodamine-conjugated Phalloidin (Thermo Fisher Scientific Inc). Glass coverslips were mounted in Vectashield mounting media (Vector Laboratories, Burlingame, CA, USA) containing diamidino-2-phenylindole (DAPI) to counterstain the nuclei. The fluorescent images were taken with Nikon Eclipse E50i microscope (Nikon, Minato, Japan). The images were taken with Nikon 10× and 40× magnification objectives, and then analysed with the ISIS software (MetaSystems, Altlussheim, Germany). In order to quantify cells with structural changes, 15 images of random fields at 10× magnification were taken per each experiment, with an average of 20 cells per image. Cells with visible stress fibres or circumferential actomyosin belt (CAB) rearrangements were counted. The percentage values were calculated.

### 2.10. Statistical Analysis

Statistical analysis and graphs were done using GraphPad Prism version 7.00 for Windows (GraphPad Software, La Jolla, CA, USA). Two-tailed χ^2^ test and Pearson correlation coefficient were used to determine the significance of the differences among covariates. For in vitro experiments, data from a minimum of three independent experiments are presented as mean ± s.d. Student’s *t*-test (two-tailed) were used to compare two groups and *p* < 0.05 was considered to be significant.

## 3. Results

### 3.1. IPMN miRNome and Transcriptome Exploration

**Figure 1:**
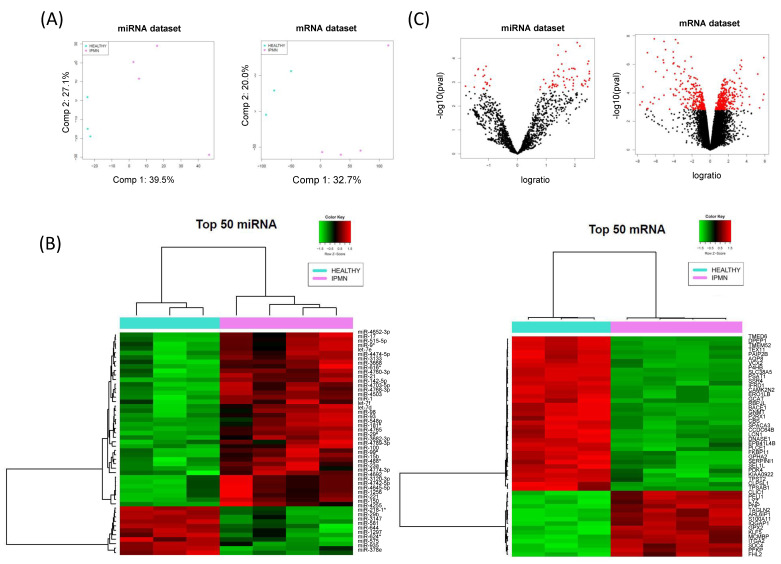
Paired miRNA and mRNA expression data in IPMN. (**A**) Principal Components Analysis plot representation of correlation matrix for miRNA (left) and mRNA (right) expression data in Healthy (*n* = 3) and IPMN (*n* = 4) pancreatic tissue samples. (**B**) Heatmaps of the top 50 differentially expressed miRNAs (left) and mRNAs (right) sorted by absolute FC value (FDR < 0.05). (**C**) The volcano plot of expression data for miRNAs (left) and mRNAs (right), red dots highlight for FDR < 0.05 and absolute FC > 2.

Analysis of miRNome and transcriptome paired data from healthy and IPMN tissues reported significant differential expression of 75 miRNAs and 562 mRNAs in IPMN versus healthy, respectively. IPMN samples and healthy pancreatic tissues clustered differently, depending either on miRNA or mRNA profiling (Figure 1A).

We reported 57 significantly upregulated and 18 significantly downregulated miRNAs in our IPMN set with respect to the healthy tissues. Several of these upregulated miRNAs have been validated by qRT-PCR in other cohorts of IPMN patients in previous studies [9,11]. On the other hand, 314 significantly upregulated and 248 significantly downregulated mRNAs were found to be significantly altered between IPMN and healthy tissues (Figure 1B). MiRNAs and mRNAs with FDR < 0.05 were selected for further exploration (Figure 1C).

### 3.2. MiRComb Filtering of Potential miRNA-mRNA Pairs Occurring in IPMN

**Figure 2:**
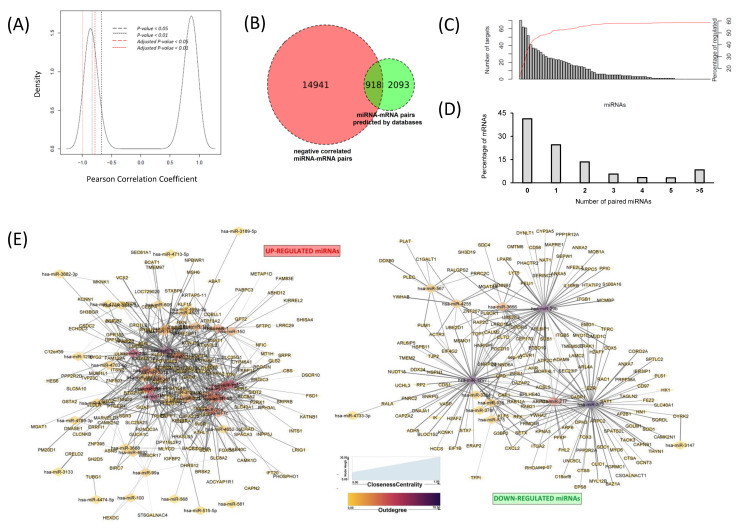
MiRNA-mRNA interactions predicted by miRComb in IPMN. (**A**) Pearson Correlation plot showing density of miRNA-mRNA pairs. Only pairs showing negative correlation and adjusted *p*-value < 0.05 were selected. (**B**) Venn diagram. Red: number of miRNA-mRNA pairs with negatively correlated expression in IPMN and healthy pancreatic tissues (adj. *p*-value < 0.05). Green: all the theoretical miRNA-mRNA pairs reported by at least 1 of the following miRNA target prediction databases: targetScan_v7.1_17, miRSVR_aug10_17, and miRDB_v5.0_21. Intersection: miRNA-mRNA pairs that fulfil both conditions. (**C**) Barplot representing the number of mRNAs targeted per each miRNA (each bar represents a miRNA). MiRNA-mRNA interactions with pval-corrected < 0.05 and predicted at least 1 time on the studied databases. Red line (and right axis) represents the percentage of deregulated mRNAs that are cumulatively targeted by the miRNAs. (**D**) Barplot representing the number of miRNAs targeting each mRNA. (**E**) Network analysis of miRNA-mRNA couples reported in IPMN. Directed networks of miRNAs and mRNAs are represented. Circle size indicates Closeness to centrality; circle colours indicate outdegree (Min.-yellow, max.–dark purple). We can see two networks corresponding to upregulated miRNAs with its downregulated pairs and vice versa.

The detected upregulation and downregulation of both miRNA and mRNA in the same dataset allow us to explore negative correlations between them. We selected the 75 and 562 significantly deregulated miRNAs and mRNAs, respectively, and computed all of the possible correlations using MiRComb package. Among 42,150 possible pairs, 19,788 were negatively correlated, and 15,859 also had an adjusted *p* value < 0.05, representing 37.6% of the total miRNA-mRNA reported combinations (Figure 2A). Subsequent filtering according to the presence in at least one miRNA target prediction database used by MiRComb led to 918 potential miRNA-mRNA couples occurring in IPMN (Figure 2B). This step enabled filtering 30.5% among all of the miRNA-mRNA predicted interactions. The couples corresponded to combinations between 330 mRNAs and 62 miRNAs, and indicated that, according to the model, 59% of significantly deregulated mRNAs in IPMN would be targeted by at least one miRNA.

Regarding the number of targets per miRNA, 29.5% of the paired miRNAs showed more than 20 targets according to our model (Figure 2C). Redundancy on miRNA targeting, when considering those targets regulated by more than 5 miRNAs, was seen in 20% of mRNAs from the above mentioned miRComb miRNA-mRNA pairs (Figure 2D). Supplementary Table S2 shows the 918 miRComb miRNA-mRNA interactions in the IPMN dataset and Supplementary Figure S1 shows miRNA-mRNA expression correlation for the 15 most significantly correlated miRNA-mRNA pairs of that table. Table 1 shows the top 45 most significant interactions with information regarding their correlation coefficients and their presence in the target prediction databases. Between them, seven were found to have correlation coefficients higher than 0.99, including miR-1297-RAB21, miR-29b-MORF4L1, miR-29b-SUB1, miR-181a-TPST2, miR-1297-DDX24, miR-21-KLB, and miR-181a-VCX2. All of the miRNAs on these highly confident couples, except miR-21, were reported to have more than 50 targets.

A total of 18 miRNAs were reported to have more than 20 target mRNAs, with miR-372, miR-1297, and miR-29a being the ones appearing in more pairs, suggesting broad regulatory functions for these miRNAs in IPMN (Supplementary Table S3). On the other hand, six mRNAs were reported to be targeted by more than 10 miRNAs (Supplementary Table S4), suggesting a strong control over those reads, according to our model. The top mRNA in this category was LRIG1, a regulator of signalling by receptor tyrosine kinases. This mRNA targeted by lots of miRNAs might be a key pathway modulator in IPMN. Network analysis of miRNA-mRNA interactions revealed two main clusters. They corresponded to downregulate d miRNAs with upregulated target mRNAs, and vice versa. MiRNA-mRNA pairs on both clusters accounted for the 95.2% of the reported significant ones, indicating that, by using our MiRComb approach, we are able to undercover highly connected molecular networks (Figure 2E).

### 3.3. Contribution of miRNA-mRNA IPMN Interactions in PDAC

We used a dataset of paired miRNA-seq and transcriptomic data of PDAC samples from our previous study in order to evaluate how miRNA-mRNA interactions reported in IPMN were represented in the PDAC context [14]. We wanted to analyse which miRNA-mRNA pairs in our IPMN samples were maintained or disappeared in PDAC and, thus, were likely to be related with tumour malignisation or maintenance.

MiRComb correlation and database prediction filtering led to 17,401 significant miRNA-mRNA pairs in the PDAC context. The intersection of both datasets unravelled 445 common miRNA-mRNA pairs in IPMN and PDAC (Supplementary Table S5), which formed by combinations between 42 miRNAs and 189 mRNAs. These couples corresponded to 48.5% and 2.6% of the miRNA-mRNA pairs reported for IPMN and PDAC, respectively.

On this final set of common miRNA-mRNA interactions, there was a decrease on the number of targets per miRNA. A total of six miRNAs were reported to have more than 20 target mRNAs (Table 2), with miR-181a being the upregulated miRNA with more targets, and miR-372 the downregulated miRNA with more targets according to the model, suggesting potential roles on the IPMN to PDAC progression. On the other hand, 1 mRNA, LRIG1, was also reported to be targeted by more than 10 miRNAs in the PDAC setting as in IPMN, which suggests high control over those reads also in the PDAC context (Supplementary Table S6).

### 3.4. In-Silico Functional Analysis of miRNA-mRNA Pairs

**Figure 3:**
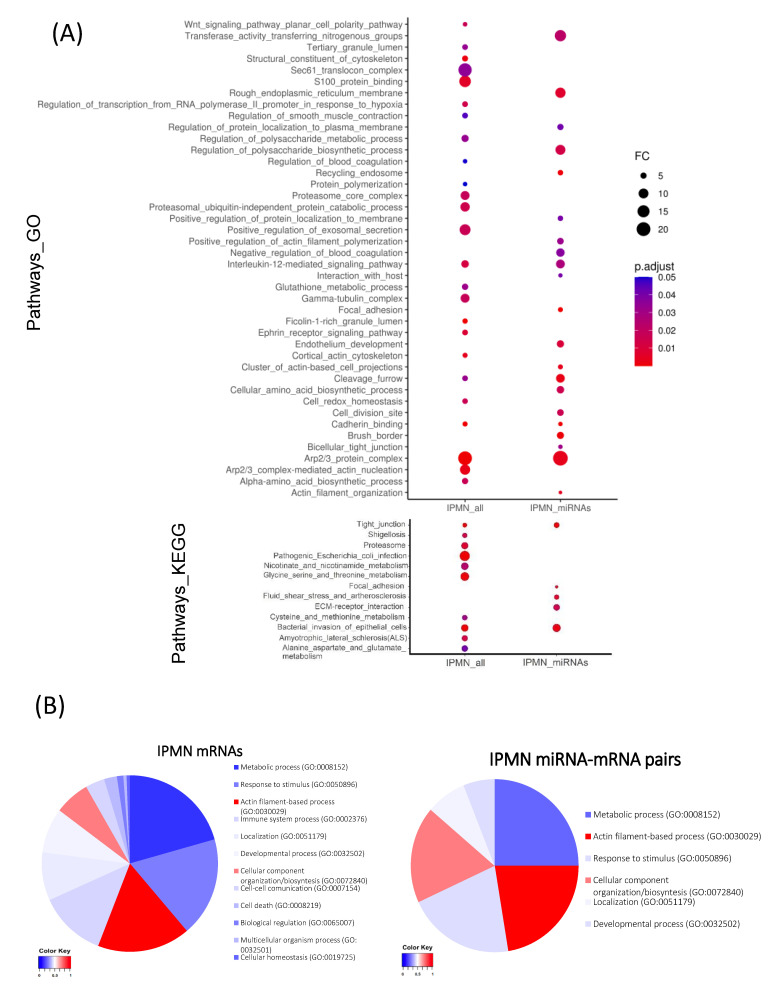
In silico functional analysis of miRNA-mRNA interactions predicted by miRComb in IPMN. (**A**) Dot plot showing comparative pathway enrichment analysis from all deregulated mRNAs in IPMN samples (IPMN_all) and from only those mRNAs paired with miRNAs in our model (IPMN_miRNAs); adj *p*-value < 0.05; FC > 4. Gene Ontology analysis (Biological process, Cellular component, Molecular function) plus KEGG pathways results are shown. (**B**) Pie charts representing functional analysis from all differentially expressed mRNAs in IPMN (left), and from only those miRNA paired mRNAs in IPMN (right). The represented data come from Gene Ontology Biological Process report. Gradient for distribution of genes related with cell structure (cytoskeleton GO:0005856, cell adhesion GO:0007155 and extracellular matrix GO:0043062), among enriched pathways. Scores correspond to the normalized proportion of cell structure proteins from our dataset in each enriched pathways. Scores: Maximum 1 (red) minimum 0 (blue).

Gene Ontology and KEGG analysis were performed in the whole IPMN transcriptomic dataset, and in miRNA-paired mRNAs of the IPMN dataset (Figure 3A). We reported significant alterations on metabolic processing of proteins and polysaccharides, cell homeostasis, cell cycle, cytoskeleton, response to cytokines, exocytosis, and different signalling pathways, including developmental programmes, such as WNT. However, the functional study of mRNAs in miRNA pairs showed a specific profile. We reported significant alterations on pathways related with cellular movement, tight junctions, cytokinesis, and pathways that are related with endothelium development and the endoplasmic reticulum. Moreover, those pathways related with actin filaments, cell anchoring, and response to IL-12, as well as some metabolic processes, were also significantly altered in IPMN when only analysing the subset of miRNA regulated genes. Detailed enrichment report can be found in Supplementary Tables S7 and S8.

The distribution of genes related with cell structure (including cytoskeleton, cell adhesion-related genes, and extracellular matrix components) among enriched pathways was mild in the transcriptomic landscape of IPMN, but became enriched in the miRNA-regulated fraction of IPMN altered genes, according to our model (Figure 3B). In the IPMN dataset, pathways with more than 50% of the enriched proteins being related with cell structure accounted for the 23.5% of the enriched categories, while, in the IPMN-miRNA set, it accounted for the 40.8%.

**Figure 4:**
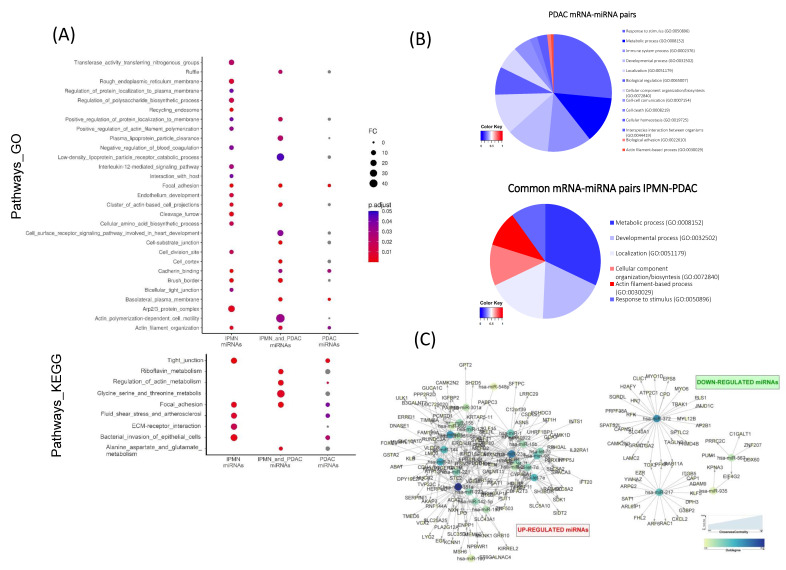
In silico functional and network analysis of common miRNA-mRNA interactions predicted by miRComb in IPMN and PDAC. (**A**) Dot plot showing comparative pathway enrichment analysis of IPMN miRNA-paired mRNAs (IPMN-miRNAs), miRNA-paired mRNAs commonly reported in IPMN and PDAC (IPMN_and_PDAC-miRNAs), and PDAC miRNA-paired mRNAs (PDAC-miRNAs) adj *p*-value < 0.05. Grey dots indicate enriched pathways with adj *p*-value ≥ 0.05. Gene Ontology analysis (Biological process, Cellular component, Molecular function) plus KEGG pathways results are shown. (**B**) Pie charts representing functional analysis of whole PDAC miRNA-paired mRNAs, and of only those miRNA-paired mRNAs commonly reported in IPMN and PDAC. Represented data come from Gene Ontology Biological Process report. Gradient for distribution of genes related with cell structure (cytoskeleton GO:0005856, cell adhesion GO:0007155 and extracellular matrix GO:0043062), among enriched pathways. Scores correspond to the normalized proportion of cell structure proteins from our dataset in each enriched pathways. Scores: Maximum 1 (red) minimum 0 (blue). (**C**) Network analysis of miRNA-mRNA couples commonly reported in IPMN and PDAC. Directed networks of miRNAs and mRNAs are represented. Circle size indicates Closeness to centrality; circle colours indicate outdegree (Min.-yellow, max.–dark blue). We can see two networks corresponding to upregulated miRNAs with its downregulated pairs and vice versa. Nodes with Closeness to centrality = 1 and Oudegree > 25 corresponded to hsa-miR-181a, hsa-miR-23a, hsa-miR-372 and hsa-miR-93.

In order to further explore the functional contribution of miRNA-mRNA interactions appearing in IPMN and conserved in PDAC, we performed a pathway enrichment analysis of the 189 mRNAs in the 445 miRNA-mRNA pairs common between IPMN and PDAC, and compared the results with the pathway enrichment analysis of mRNAs from the whole mRNA-miRNA pairs in PDAC (Figure 4A). We observed an overrepresentation of pathways that were related with actin cytoskeleton and extracellular matrix interaction through tight junctions in the set of common interactions between IPMN and PDAC, with respect to those pathways enriched in the miRNA regulated mRNAs according to the model in the IPMN or PDAC datasets, individually. Strikingly, only pathways that were related with cell structure were significant in all three datasets. We also reported pathways related with several metabolism processes. Detailed enrichment report for common miRNA regulated mRNAs in IPMN and PDAC can be found in Supplementary Table S9.

When focusing on cell structure genes, it was observed that enriched pathways in the whole miRNA-related mRNAs in the PDAC dataset had a low percentage of structural genes. In fact, in PDAC miRNA-paired genes, pathways with more than 50% of the enriched proteins being related with cell structure, such as actin-filament processes or cellular component organization, accounted for the 1.7% of the enriched categories, while, in the set of IPMN and PDAC common miRNA-paired genes, it accounted for the 22.7% (Figure 4B). These results suggested hierarchy over these processes, for miRNA-mRNA pairs that were conserved in the IPMN and PDAC context.

Network analysis showed two clearly differentiated networks, corresponding to downregulated miRNAs with upregulated target mRNAs, and vice versa (Figure 4C). Nodes with highest closeness to centrality and outdegree values corresponded to main regulators of the sets, with miR-181a being the node with highest values in the up-regulated miRNAs network, and miR-372 the main node in the principal network of down-regulated miRNAs.

### 3.5. Validation of Functionally Relevant miRNA-mRNA Negative Correlations for miR-181a and miR-372 by RT-qPCR in an Independent Cohort

**Figure 5:**
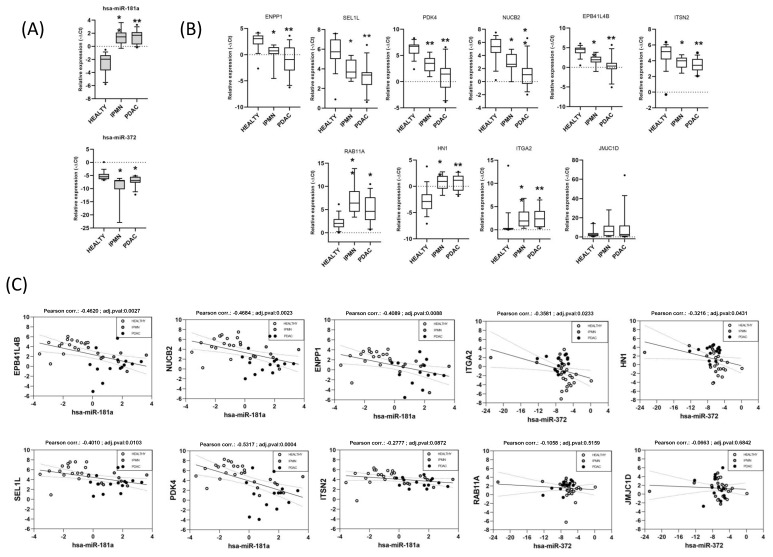
qRT-PCR expression analysis of selected miRNA-mRNA pairs in pancreatic patient samples. (**A**) Boxplots for miRNA expression (miR-181a and miR-372) in Healthy (*n* = 17), IPMN (*n* = 9) and PDAC (*n* = 14) pancreatic samples, samples with deviation from the mean >5% are represented as black dots. (**B**) Boxplots for mRNA expression of selected potential targets related with metabolic alterations (NUC2B, SEL1L, ENPP1, PDK4 and JMJD1C, RAB11A) and with cytoskeletal-related pathways (EPB41L4B, ITSN2 and ITGA2, HN1), respectively, in Healthy (*n* = 17), IPMN (*n* = 9) and PDAC (*n* = 14) pancreatic samples, samples with deviation from the mean >5% are represented as black dots (**C**) Correlation plots of miRNA versus mRNA expression values measured by qRT-PCR (−ΔCt). * *p* value < 0.05 ** *p* value < 0.001.

To confirm whether some of the main miRComb miRNA-mRNA pairs were also occurring in another cohort of patients, we analysed the expression of 10 selected pairs from the main miRNA nodes in IPMN-PDAC common interactions by qRT-PCR in 40 independent pancreatic samples (14 PDAC, nine IPMN, 17 Healthy). MiR-181a (Assay ID 000480) and miR-372 (Assay ID 000560) were chosen for validation due to its central role in the obtained networks. The analysed targets (NUC2B, assay ID Hs01093821_g1; SEL1L, Hs01071398_m1; ENPP1, Hs01054040_m1; PDK4, Hs01037712_m1; EPB41L4B, Hs00219582_m1; ITSN2, Hs01049908_m1 and ITGA2, Hs00158127_m1; HN1, Hs00602957_m1; JMJD1C, Hs00934835_m1; RAB11A, Hs00366449_g1) were chosen according to its correlation coefficient and its presence in enriched pathways with high content of cytoskeletal and actin related proteins or related with metabolic alterations, respectively, as these were the most outstanding enriched programmes. The −ΔCt values of miRNAs and mRNAs from each sample in the set were analysed for negative correlation (*p* < 0.05). We confirmed significant upregulation and downregulation of miR-181a and miR-372, respectively, in the IPMN and PDAC samples versus healthy (Figure 5A). Conversely, a significant downregulation of the selected pairs for miR-181a and upregulation of those for miR-372 was also confirmed in IPMN and PDAC samples versus healthy (Figure 5B). We validated significant negative expression correlations of miR-181a with NUC2B, SEL1L, ENPP1, PDK4, EPB41L4B, and of miR-372 with ITGA2 and HN1 (Figure 5C).

### 3.6. MiR-181a Staining Is Increased in Pancreatic Ductal Cells from IPMN and PDAC

**Figure 6:**
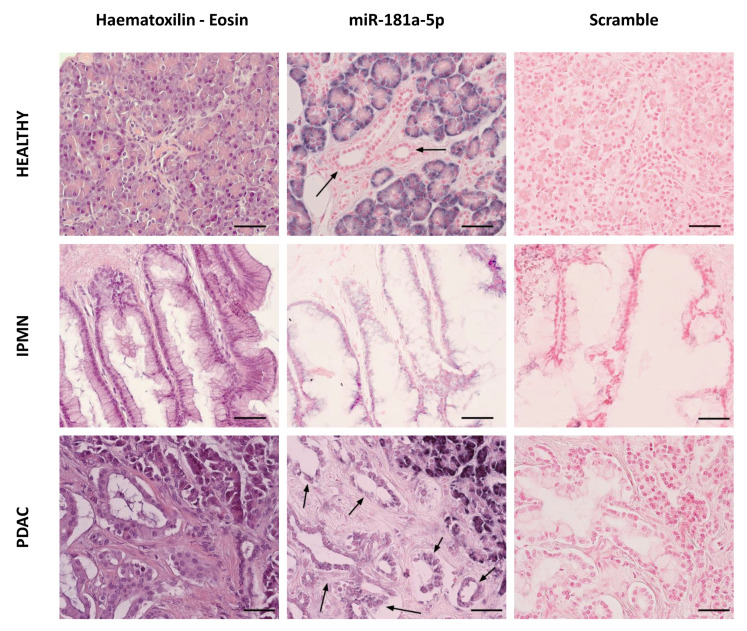
In situ hybridization of miR-181a-5p in Healthy, IPMN, and PDAC pancreatic tissues. From left to right: Representative haematoxylin-eosin staining of the analysed tissues. Representative images of LNA-miR-181a-5p probe and Scrambled miR-control probe staining. Arrows highlight ductal cells. High miR-181a-5p expression is shown as blue/purple, low expression is shown as light pink. Representative images from Healthy (*n* = 4), IPMN (*n* = 3) and PDAC (*n* = 4) pancreatic tissues. Magnification: 40×. Scale bars size 50 μm.

In order to evaluate the patterns of expression of miR-181a in pancreatic human samples, in situ hybridization was performed in PDAC, IPMN, and normal pancreas. The ductal cells in the normal pancreatic tissue showed non-detectable staining of miR-181a. In contrast, miR-181a expression increased in transformed ducts from IPMN and PDAC (Figure 6). Acinar cells showed strong positive miR-181a staining in both normal and tumoral tissues, and positive miR-181a staining was also detected in some tumour-associated fibroblasts and inflammatory infiltrates from PDAC samples.

### 3.7. Negative Correlation between miR-181a Expression and Its Potential Targets in Pancreatic Cancer Cell Lines

**Figure 7:**
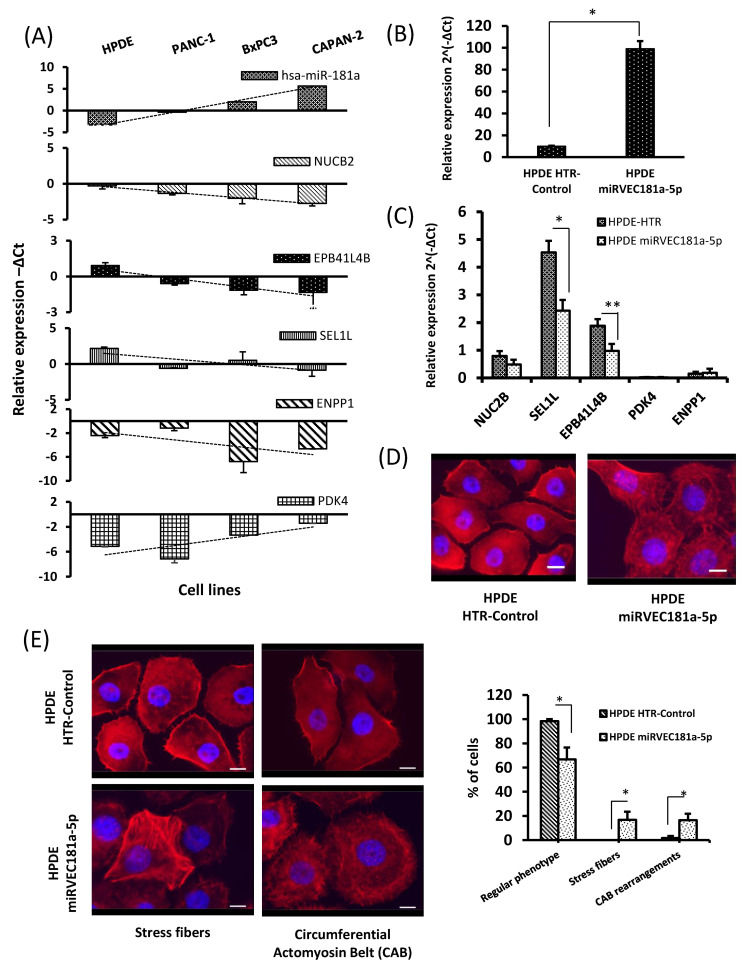
Functional analysis of potential miR-181a targets in pancreatic cellular models. (**A**) Pannel barplot. Expression of miR-181a and its potential targets analysed by qRT-PCR in pancreatic cell lines HPDE, PANC-1, BxPC3, and CAPAN-2. (**B**) qRT-PCR expression of miR-181a in HPDE cell lines transduced with HTR-Control or miRVEC181a-5p, an overexpression model. (**C**) qRT-PCR expression of potential miR-181a targets in HPDE HTR-Control and HPDE miRVEC181a-5p cell lines. (**D**) Representative images of phalloidin-rhodamine staining of HPDE miRVEC181a-5p and HPDE HTR-Control cell lines. Actin filaments are stained in red, nucleus are stained in blue (DAPI). Images are taken at 40× magnification, scale bar 100 μm. (**E**) The representative images of cells showing regular phenotype, stress fibres, or circumferential actomyosin belt (CAB) rearrangements in HPDE HTR-Control and HPDE miRVEC181a-5p cell lines (left) and cell quantification (right). The data of *n* = 3 independent experiments. *p* value < 0.05 (*), *p* value < 0.01(**).

In our previous study, miR-181a was validated as a potential biomarker for early detection of PDAC, already detectable in IPMN patients [9]. In order to further confirm the potential contribution of the above-mentioned miR-181a-target pairs in pancreatic ductal cell transformation, we analysed their expression in a set of pancreatic cell lines, including normal ductal cells, HPDE, and pancreatic cancer cells (PANC-1, BxPC-3, and CAPAN-2). A negative expression correlation (*p* < 0.05) between miR-181a and its potential targets was also reported for NUC2B, SEL1L, EPB41L4B, and ENPP1 (Figure 7A). We could not perform the same analysis with miR-37, because its expression in the analysed cell lines was undetectable by qRT-PCR; in fact, this miRNA is extremely downregulated in pancreatic cancer.

### 3.8. Pancreatic Ductal Cells Overexpressing miR-181a Show Alterations of the Actin Cytoskeleton Organization

In order to further explore the contribution of the miR-181a overexpression seen in IPMN and PDAC through the suggested pathways, we generated a cellular model mimicking early events of pancreatic ductal neoplasia according to our results by constitutively overexpressing miR-181a in the immortalized human pancreatic ductal cell line HPDE. We reported a stable upregulation of miR-181a in HPDE miRVEC181a-5p model with respect to HPDE HTR-Control (Figure 7B). An evaluation of the previously mentioned miR-181a mRNA pairs by qRT-PCR in this cell model showed a significant downregulation of SEL1L and EPB41L4B (Figure 7C). It is important to consider that the basal levels of PDK4 and ENPP1 from the HPDE HTR-Control cell line were so low that it was difficult to observe a miRNA-induced downregulation of these genes in this model.

Because actin cytoskeleton organization was one of the main pathways showing a increase of representation in IPMN-PDAC miRNA regulated genes and most of the miR-181a miRComb targets are functionally related to this pathway, we performed phalloidin staining in this cell model to test the effect of miR-181a upregulation on actin cytoskeleton structures. Fluorescent microscopy images showed significant differences between HPDE cells overexpressing miR-181a and the respective controls regarding cell shape and actin organization (Figure 7D). Moreover, cells overexpressing miR-181a had diffuse cell junctions. A significant increase in the number of cells with defined contractile structures, such as stress actin fibres and rearrangements in the circular actomyosin belt (CAB), was reported (Figure 7E), indicating an active role of miR-181a on tuning actin cytoskeleton structure.

### 3.9. Immunohistochemical Staining of EPB41L4B and SEL1L in Healthy, IPMN and PDAC Tissues

**Figure 8:**
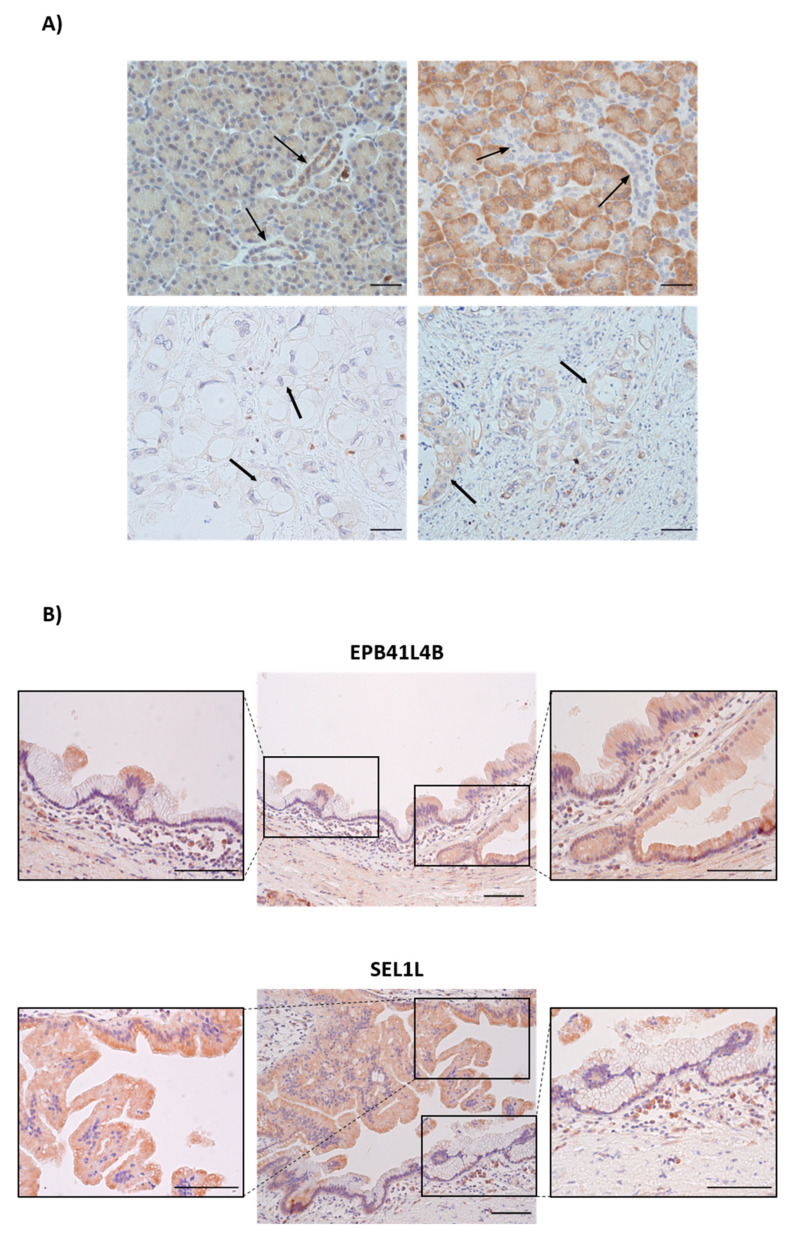
Immunohistochemistry staining of EPB41L4B and SEL1L in Healthy, IPMN and PDAC tissues. (**A**) EPB41L4B and SEL1L staining in healthy pancreatic tissue and PDAC. Arrows highlight ductal structures. Magnification: 40×. Scalebars size 50 μm. (**B**) Panel showing staining of EPB41L4B and SEL1L in IPMN. The central panel images were taken at 20× magnification. Representative images from Healthy (*n* = 4), IPMN (*n* = 3) and PDAC (*n* = 4) pancreatic tissues. Lateral detail images were taken at 40× magnification. Scale bars size 100 μm in all of the images of the panel.

Regarding EPB41L4B, staining was mainly detected in ductal cells and blood vessels from normal pancreatic tissues, although some acinar background was also detected. Concordantly with our results, the staining from ductal cells disappeared in oncogenic cells from PDAC samples (Figure 8A). Moreover, a positive signal was detected in some tumour-associated fibroblasts and some inflammatory infiltrates in PDAC samples. However, IPMN lesions showed heterogeneous pattern of EPB41L4B staining, with some parts of the cyst layer showing positive staining and some parts negative (Figure 8B).

Regarding SEL1L, strong positive staining was mainly detected in acinar cells from normal pancreatic tissues confirming previously published results, [16] and to a lower extent in ductal cells (Figure 8A). PDAC sections showed a slightly increase in SEL1L staining in transformed ductal cells, suggesting that other factors or miRNAs apart from miR-181a may be regulating SEL1L expression in this context. Similarly to EPB41L4B, the IPMN lesions showed a heterogeneous pattern of SEL1L staining, with some parts of the cyst layer showing positive staining and some parts negative (Figure 8B).

## 4. Discussion

By combining in-silico analysis of miRNome and transcriptome data from healthy pancreatic tissues and IPMN or PDAC tumors via MiRComb software [13], plus qRT-PCR analysis in an independent set of samples, we have found that two commonly deregulated miRNAs (miR-181a and miR-372) in IPMN and PDAC are, in both contexts, negatively correlated with mRNAs encoding actin related proteins (EPB41L4B, ITGA2, and HN1) and metabolism related effectors (NUC2B, SEL1L, ENPP1, and PDK4). MiRComb has been demonstrated to be a useful tool in terms of filtering of potential miRNA-mRNA pairs in a specific setting [14], although the validation of reported pairs in alternative cohorts of patients is always discussed to be required. In this study, an independent cohort validation was performed in order to further confirm relevant miRNA-target regulations in IPMN-PDAC progression.

IPMNs are potential risk factors for the development of PDAC, when showing high-grade dysplasia [17]. In the IPMN context, actin accumulation has been reported to be correlated with an increment of histological grade of the lesions [18], meaning that the alteration of the actin cytoskeleton is an early occurring event, being linked with dysplasia. Furthermore, direct impact of actin cytoskeleton alterations on metabolic impairment and endoplasmic reticulum (ER) homeostasis is known to happen in eukaryotic cells and tumours [19,20]. Data from our study link miRNA alteration in IPMN and PDAC with these processes, thus linking the impairment of miRNA expression with actin related pathways and tumour progression.

However, and although we have used an independent set of samples to validate some results, we have to take that the number of human samples used is still limited into account, and the results from this study should be considered as pilot. Moreover, surgical samples used for the analysis may include different cell types, which also represent a caveat of the study. The analysis of correlations between miR-181a and the mentioned targets in a set of pancreatic cancer cell lines with different basal miR-181a expression allowed us to discern which targets were likely to take place in tumoral cells. Although the performances of NUCB2, EPB41L4B, SEL1L, and ENPP1 were quite consistent, we observed an uneven distribution in PDK4 expression across these cell lines, meaning that the relation between PDK4 expression and miR-181a in tumour cells may not be causal, and the contribution of these pairs might come from other cell types, such as cancer associated fibroblasts or immune infiltrates.

In situ hybridization staining of miR-181a showed that healthy pancreatic ductal cells express low levels of miR-181a, whereas IPMN lesions and PDAC show higher levels of expression of this miRNA. This fact suggests a potential effect of miR-181a overexpression in the tumorigenic transformation of ductal cells in PDAC. Regarding the potential role of miR-181a sustaining IPMN malignancy, we analysed the effect of constitutive miR-181a overexpression in an immortalized pancreatic ductal cell line, HPDE. The quantification of expression indicated a significant downregulation of EPB41L4B, a modulator of the circumferential actomyosin belt in epithelial cells [21], and SEL1L, a mediator of ER-associated degradation [16] and adhesion during pancreatic development [22]. PDK4 and ENPP1 did not show significant downregulation that was mainly due to the low basal levels of expression of these genes in the normal HPDE cells. MiR-181a-mediated regulation of some of these reads may occur in other cell types of the tumour, or could merely be a secondary effect. Concordantly to the observed downregulation of EPB41L4B and SEL1L in HPDE cells overexpressing miR-181a, we observed alterations on actin structure that were related to an increment on the number of cells with wide stress fibres and polygonal shape, plus an enrichment of cells with re-organized circumferential actomyosin belt. The presence of stress fibres has been reported to be a characteristic of unfolded protein response, in different cell types [23], with endoplasmic reticulum homeostasis breakage being one of the functional implications of SEL1L deregulation. In our functional enrichment analysis of common miRNA-mRNA pairs in IPMN and PDAC, endoplasmic reticulum stress was one of the main altered metabolic pathways. Furthermore, EPB41L4B’s active role on circumferential actomyosin belt organization is consistent with the altered actin process pathway in our model. These facts reinforce the idea that the deregulation of miR-181a is tuning the expression of some genes involved in cell structure remodelling during IPMN establishment and in PDAC. MiRComb also reported other potential miRNA regulators for these proteins. As an example, miR-155 has been described to participate in the regulation of SEL1L expression in PDAC [24], and it has also been reported to be a potential pair for SEL1L in our dataset of IPMN-PDAC common miRNA-mRNA pairs too.

Regarding the relevance of miR-181a in PDAC initiation, it was shown that miR-181ab1 cluster tunes this process in Ptf1aCre/+, KrasLSLG12D/+ Trp53fl/fl, and Mir181ab1−/− mice [25]. Mir181ab1 knock-out developed less PanIN lesions, and it had less aggressive pancreatic tumours than controls. Consistently, one of the conclusions that can be drawn from our study is that miR-181a could also contribute to PDAC evolution from IPMN. In the referred study, the miR-181ab1 cluster was related to proteins that were involved in cell structure, results that are in concordance with the outputs of ours. Therefore, we can speculate that miR-181a is related with the progression to PDAC from both PanIN and IPMN lesions. Moreover, it has been described that miR-181a is also involved in PDAC progression by inducing epithelial-mesenchymal transition and pancreatic cancer cell migration, thus reinforcing the idea that this miRNA is playing important roles in pancreatic carcinogenesis [26,27,28].

Although we have mainly focused on some miR-181a and miR-372 pairs, the results that were obtained in the present study allow for discussing the functional role of several other miRNAs in the context of IPMN and PDAC. In that sense, miR-23a or miR-93 are other interesting miRNAs in that context. In fact, we had previously studied the functional role of miR-93 in PDAC, demonstrating the implication of this miRNA in the regulation of cytoskeleton structure [29]. These results are in concordance with the present study, and they reinforce the concept that miRNAs already deregulated in IPMN could be related with cell structure modulation. This suggested link between miRNAs and regulation of cell structure could imply potential roles of miRNAs as biomarkers of cell structure alteration status in PDAC, with potential applications in tracking sensitivity to cell structure-related anticancer drugs.

Nevertheless, it is important to consider that, although we have been able to see and validate significant negative correlations between miRNA-mRNA reported pairs, we cannot assure that all of them are direct targets, and experimental confirmation of the direct interactions via luciferase reporter assays would be required. However, the aim of this study was roughly revealing the miRNA network around IPMN and IPMN-PDAC progression more than describing deeply those directly related targets. In that sense, what we can confirm is a functional relation between miR-181a and SEL1L and EPB41L4B in pancreatic ductal cells, either direct or indirect, as demonstrated in our overexpression model. The fact that the initial steps during the filtering process include prediction databases, while taking 3’UTR complementarity to miRNA into account, makes us think that it is highly probable that these are direct targets. Despite the caveats, miRComb analysis considerably reduces the number of potential miRNA target interactions, and enrichment analysis of those pairs highlights pathways whose alteration can be detected in vitro via tuning of miRNA expression.

MiRNAs are being described as modulators of cell plasticity in many contexts. It was demonstrated that an efficient miRNA-based regulation is only affordable when relying on high turnover rate protein targets [30]. Using our target-filtration strategy, we have been able to detect a set of proteins, likely to be regulated by miRNAs in premalignant lesion as IPMN, enriched in processes that are related with rapid turnover, such as dynamic cytoskeletal structures [31,32], which may influence many processes, such as metabolic responses [33,34]. With these results, we could hypothesize that we are focusing precisely on those pathways in which miRNAs are mechanistically playing a major role. This fact would mean that, in the context of IPMN, early altered miRNA-regulated pathways that are maintained in PDAC are tuning dynamic processes that push cells towards a pro-oncogenic state.

In this study, we unravel the potential implications of miRNA alteration in the structural and metabolic changes occurring in pancreatic cells during IPMN establishment and highlight those that are maintained in PDAC. These results offer new insights on the roles of miRNAs in the IPMN-to-PDAC progression.

## 5. Conclusions

By combining miRNA-mRNA pancreatic expression plus negative correlation filtering, we have unveiled multiple miRNA-mRNA couples that may be ocurring in pancreatic cells during IPMN establishment and highlighted those that may be involved in IPMN-to-PDAC progression. Results have pointed out miR-181a as a master regulator involved in this process, by regulating actin cytoskeleton alterations and cellular stress.

## Figures and Tables

**Table 1:** Top 45 miRNA-mRNA pairs in IPMN (sorted by adjusted *p*-value). Each miRNA-mRNA pair has pval-corrected < 0.05 and appear at least 1 times in the following databases: targetScan_v7.1_17, miRSVR_aug10_17, miRDB_v5.0_21.

miRNA	mRNA	Cor	Adj.*p*val	FC.miRNA	FC.mRNA	Dat.Sum
hsa-miR-1297	RAB21	−1.00	5.14 × 10^−3^	−2.27	2.03	2
hsa-miR-29b	MORF4L1	−1.00	5.14 × 10^−3^	−2.21	2.22	2
hsa-miR-29b	SUB1	−0.99	1.08 × 10^−2^	−2.21	2.06	2
hsa-miR-181a	TPST2	−0.99	1.08 × 10^−2^	5.67	−34.93	2
hsa-miR-1297	DDX24	−0.99	1.08 × 10^−2^	−2.27	2.39	1
hsa-miR-21	KLB	−0.99	1.08 × 10^−2^	5.45	−1.70	1
hsa-miR-181a	VCX2	−0.99	1.08 × 10^−2^	5.67	−30.12	1
hsa-miR-144	CEBPA	−0.98	1.11 × 10^−2^	2.64	−3.33	1
hsa-miR-21	ERO1LB	−0.98	1.11 × 10^−2^	5.45	−11.93	1
hsa-miR-144	HERPUD1	−0.98	1.11 × 10^−2^	2.64	−3.70	1
hsa-miR-29a	UHRF1BP1	−0.98	1.11 × 10^−2^	3.29	−2.26	1
hsa-miR-29b	S100A16	−0.98	1.11 × 10^−2^	−2.21	11.23	1
hsa-miR-181a	PDK4	−0.98	1.11 × 10^−2^	5.67	−9.62	2
hsa-miR-21	VGLL2	−0.98	1.15 × 10^−2^	5.45	−1.52	1
hsa-miR-4760-3p	TRHDE	−0.98	1.15 × 10^−2^	4.22	−5.57	1
hsa-miR-144	FAM129A	−0.98	1.16 × 10^−2^	2.64	−9.69	1
hsa-miR-4255	YWHAB	−0.97	1.16 × 10^−2^	−1.90	2.53	1
hsa-miR-1297	SETX	−0.97	1.16 × 10^−2^	−2.27	1.89	1
hsa-miR-29b	ZNF207	−0.97	1.16 × 10^−2^	−2.21	1.84	1
hsa-miR-29b	ARPC5	−0.97	1.16 × 10^−2^	−2.21	2.24	1
hsa-miR-29b	HNRNPF	−0.97	1.16 × 10^−2^	−2.21	2.78	2
hsa-miR-181a	SLC25A25	−0.97	1.16 × 10^−2^	5.67	−1.90	2
hsa-miR-29b	CTNNB1	−0.97	1.16 × 10^−2^	−2.21	1.96	1
hsa-miR-1297	EIF1B	−0.97	1.16 × 10^−2^	−2.27	1.74	1
hsa-miR-935	DENND6A	−0.97	1.18 × 10^−2^	−2.77	2.04	1
hsa-miR-3666	SUB1	−0.97	1.22 × 10^−2^	−3.49	2.06	1
hsa-miR-221	HDLBP	−0.97	1.22 × 10^−2^	3.85	−2.50	1
hsa-miR-1	SYBU	−0.97	1.22 × 10^−2^	2.35	−7.97	1
hsa-miR-29b	TOPORS	−0.97	1.22 × 10^−2^	−2.21	1.72	1
hsa-miR-29b	LPAR6	−0.97	1.22 × 10^−2^	−2.21	5.41	1
hsa-miR-181a	IFRD1	−0.97	1.22 × 10^−2^	5.67	−6.83	1
hsa-miR-181a	VGLL2	−0.97	1.22 × 10^−2^	5.67	−1.52	1
hsa-miR-15b	CYP46A1	−0.97	1.22 × 10^−2^	2.91	−1.59	1
hsa-miR-181a	ERO1LB	−0.97	1.22 × 10^−2^	5.67	−11.93	1
hsa-miR-378e	CPD	−0.97	1.22 × 10^−2^	−2.28	4.56	1
hsa-miR-606	SEL1L	−0.97	1.22 × 10^−2^	1.74	−5.33	1
hsa-miR-1297	ATP2C1	−0.97	1.22 × 10^−2^	−2.27	1.95	1
hsa-miR-372	CLIC1	−0.97	1.22 × 10^−2^	−1.95	4.52	1
hsa-miR-3133	CRELD2	−0.97	1.22 × 10^−2^	5.56	−3.67	1
hsa-miR-21	IFRD1	−0.97	1.22 × 10^−2^	5.45	−6.83	1
hsa-miR-1297	BHLHE40	−0.96	1.23 × 10^−2^	−2.27	5.95	2
hsa-miR-181a	TMED6	−0.96	1.23 × 10^−2^	5.67	−71.29	1
hsa-miR-181a	RNF144A	−0.96	1.23 × 10^−2^	5.67	−3.31	1
hsa-miR-144	VLDLR	−0.96	1.23 × 10^−2^	2.64	−5.62	2
hsa-miR-29b	MCMBP	−0.96	1.23 × 10^−2^	−2.21	2.73	1

**Table 2:** Top 10 miRNA with more targets in common IPMN-PDAC miRNA-mRNA pairs. Each miRNA-mRNA pair has *p*val-corrected < 0.05 and appears at least 1 time in the following databases: tar-getScan_v7.1_17, miRSVR_aug10_17, miRDB_v5.0_21. Grey boxes correspond to up-regulated MiRNAs in IPMN/PDACvsH, white boxes correspond to downregulated miRNAs in IPMN/PDACvsH.

miRNA	# Targets	Cum%	Targets (Top20)
**hsa-miR-181a**	46	24.33	VCX2, PDK4, SLC25A25, IFRD1, VGLL2, ERO1LB, TMED6, RNF144A, AKAP7, GPR155, PSAT1, LMO3, CYP46A1, CBFA2T3, FKBP11, NUCB2, EPB41L4B, SERPINI1, SEL1L, LYG2
**hsa-miR-23a**	32	31.74	ZNF503, SLC43A1, SYBU, MCFD2, ARHGAP18, KIAA0922, KPNA7, SEC63, GATM, SEL1L, LRIG1, ACAT1, SH3BGR, PDCD4, PDK4, ASNS, GCAT, HRASLS5, KLF11, SLC5A10
**hsa-miR-372**	29	33.33	CLIC1, ATP2C1, CAPNS1, MYL12B, JMJD1C, SPTLC2, FRMD4B, ITGA2, SQRDL, TAGLN2, LAMC2, MYO6, TOX3, CPD, PLS1, RFK, PGRMC1, PFKP, CAMK2N1, MYO1D
**hsa-miR-93**	28	33.86	KIAA0922, VLDLR, FAM129A, PTGER4, KCNJ16, LMO3, CAMK2N2, ERO1LB, CDH4, ATXN7L2, ERRFI1, PAIP2B, KLB, EPB41L4B, IFRD1, KLF11, GUCA1C, SLC16A10, MUM1L1, KPNA7
**hsa-miR-21**	24	35.98	KLB, ERO1LB, VGLL2, IFRD1, FOXO3, HERPUD1, PAIP2B, LMO3, HRASLS5, SLC16A10, BTG2, DPP10, RNF144A, RUNDC3A, ITSN2, ACAT1, AKAP7, SERPINI1, DPY19L2P2, SEC63
**hsa-let-7e**	21	37.57	SLC5A2, KIAA0922, CBFA2T3, CYP46A1, SDK1, ATXN7L2, SPACA3, HDLBP, TRHDE, HRASLS5, SIDT2, FKBP11, TPST2, P2RX1, TTN, LRIG1, RAB40C, KPNA7, BTG2, SLC8A2
**hsa-miR-217**	20	38.01	ARPC2, CAP1, G3BP2, FHL2, PFKP, SAT1, CXCL2, EZR, ADAM9, YWHAZ, RAC1, DPH3, SEP7, TOX3, SEP10, KLF5, ARL6IP1, RAB11A, ITGB5, ARF6
**hsa-miR-144**	20	38.01	HERPUD1, FAM129A, VLDLR, NUCB2, PTGER4, TTN, PDK4, VGLL2, KLB, LMO3, DNASE1, GPR155, KLF11, LOC729020, SEL1L, STC2, DPP10, ITSN2, TIMM8A, PPP2R2D
**hsa-miR-98**	18	39.07	KIAA0922, CBFA2T3, EPB41L4B, CYP46A1, SLC5A2, TTN, SDK1, TRHDE, INPP5J, HRASLS5, P2RX1, IFT20, RPH3AL, HDLBP, KPNA7, LRIG1, SLC8A2, RAB40C
**hsa-miR-1**	16	40.3	SYBU, ARHGAP18, TRHDE, CYP46A1, KRTAP5-11, KLF11, PABPC3, EPB41L4B, PDCD4, MT1H, FKBP11, LRRC29, SFTPC, KLF15, IFRD1, SEC63

#: number.

## Data Availability

Data is contained within the article or supplementary material.

## Supplementary Materials

The following are available online at https://www.mdpi.com/article/10.3390/cancers13102369/s1, Supplementary Table S1: Clinicopathological characteristics of individuals included in the study, Supplementary Table S2: 918 miRNA-mRNA pairs in IPMN (sorted by adjusted p-value) that have: pval-corrected < 0.05 and appear at least 1 times in the following databases: targetScan_v7.1_17, miRSVR_aug10_17, miRDB_v5.0_21, Supplementary Table S3: Top 10 miRNA with more targets in IPMN, Supplementary Table S4: Top 10 mRNA with more miRNAs targeting them in IPMN, Supplementary Table S5: 445 miRNA-mRNA common pairs in IPMN and PDAC (sorted by adjusted p-value) that have: pval-corrected < 0.05 and appear at least 1 times in the following databases: targetScan_v7.1_17, miRSVR_aug10_17, miRDB_v5.0_21, Supplementary Table S6: Top 10 mRNAs with more miRNAs targeting them in common IPMN-PDAC miRNA-mRNA pairs, Supplementary Table S7: Gene Ontology Enrichment Analysis of 562 derregulated mRNAs in IPMN_vs_Healthy samples, Supplementary Table S8: Gene Ontology Enrichment Analysis of 330 mRNAs in miRNA-mRNA pairs in IPMN samples, Supplementary Table S9: Gene Ontology Enrichment Analysis of 189 derregulated mRNAs in miRNA-mRNA pairs common in IPMN-PDAC samples, and Supplementary Figure S1: Plot of 15 top correlations, sorted by adjusted p-value. Databases used: targetScan_v7.1_17, miRSVR_aug10_17, miRDB_v5.0_21 (each miRNA-mRNA pair appears at least 1 time).

## Abbreviations

FC: Fold-change; HPDE: human pancreatic ductal epithelial cells; IPMN: Intraductal Papillary Mucinous Neoplasm; miRNA or miR: microRNA; PDAC: pancreatic ductal adenocarcinoma.
