# Supplementary material for: MicroRNAs Deregulated in Intraductal Papillary Mucinous Neoplasm Converge on Actin Cytoskeleton-Related Pathways That Are Maintained in Pancreatic Ductal Adenocarcinoma
Source: Cancers (Basel). 2021 May 14;13(10):2369. doi: 10.3390/cancers13102369 (PMC8155860; doi:10.3390/cancers13102369)
Supplement: Supplementary file 1 [file cancers-13-02369-s001.zip › Supplementary_Figure_1.pptx]

## Slide 1
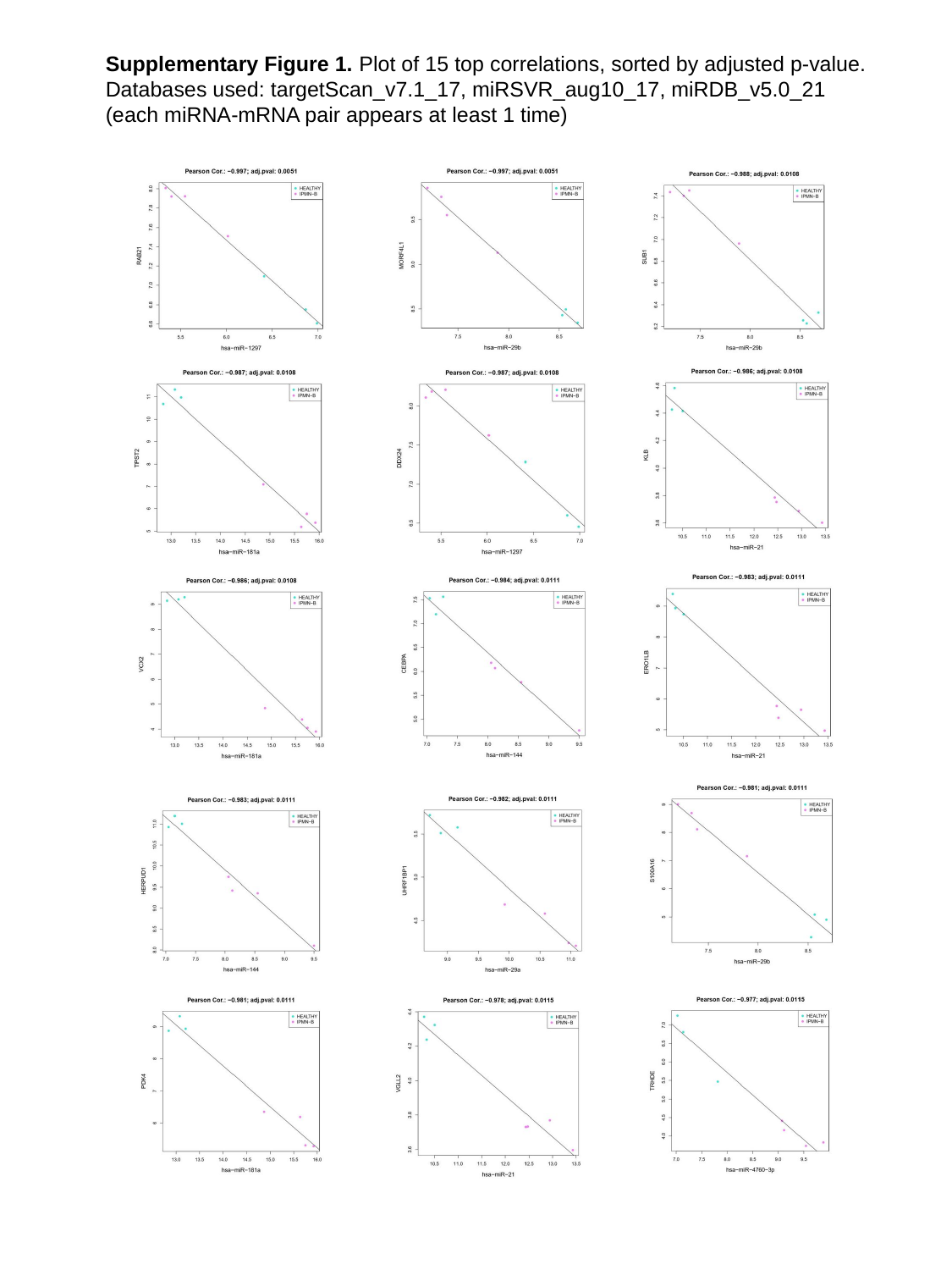

Supplementary Figure 1. Plot of 15 top correlations, sorted by adjusted p-value. Databases used: targetScan_v7.1_17, miRSVR_aug10_17, miRDB_v5.0_21 (each miRNA-mRNA pair appears at least 1 time)
